# Trends in metabolic dysfunction in polycystic ovary syndrome: a bibliometric analysis

**DOI:** 10.3389/fendo.2023.1245719

**Published:** 2023-08-28

**Authors:** Yan Xu, Zhiqun Cao, Ting Chen, Jian Ren

**Affiliations:** ^1^College of Traditional Chinese Medicine, Shandong University of Traditional Chinese Medicine, Jinan, China; ^2^The First Clinical Medical College of Shandong University of Traditional Chinese Medicine, Jinan, China

**Keywords:** polycystic ovary syndrome, metabolism, VOSviewer, CiteSpace, bibliometric analysis

## Abstract

Polycystic ovary syndrome (PCOS) is a very common chronic disease and causes reproductive disorders in women of childbearing age worldwide. The cause of metabolic dysfunction in PCOS is unknown, and there is a lack of systematic bibliometric analysis for this disease. This study included 3,972 articles on metabolic dysfunction in PCOS published from 2012 to 2021. We applied the VOSviewer and the CiteSpace scientometric analysis software to analyze the data regarding the publication of the articles, countries, authors, institutions, scientific categories, cited journals, and keywords. Through this analysis, we determined the research efforts and their developing trends and anticipated the progress in understanding PCOS-related metabolic dysfunction.

## 1 Introduction

Polycystic ovary syndrome (PCOS) is a common reproductive and endocrine disease affecting 6–10% of women of reproductive age worldwide (1). This syndrome is mostly characterized by menstrual disorder, infertility, abnormal increase in the level of androgen, and a polycystic ovary (2). Many PCOS patients have metabolic disorders, including insulin resistance, obesity, and abnormal lipid metabolism (3). The current difficulty is that the detailed pathophysiological mechanisms of PCOS are unknown (4–6).

In particular, it is important to note that elucidating the intricate relationship between the gonadotropin pathway and steroidogenesis in PCOS is critical to understanding the underlying pathophysiological mechanisms of this disorder. The hypothalamic defect prevalent in PCOS patients can cause abnormal signaling from the hypothalamus to the pituitary gland and lead to the release of excessive gonadotropin-releasing hormone (GnRH). This increases the frequency at which luteinizing hormone (LH) is released and decreases the secretion of follicle-stimulating hormone (FSH) and abnormal signaling in the pathways between them. Therefore, most PCOS patients have an increased LH/FSH ratio during ovulation (7, 8). PCOS metabolic dysfunction is related to hyperandrogenemia caused by neuroendocrine dysfunction of the hypothalamic-pituitary-ovarian axis. Additionally, hyperandrogenemia and infertility caused by PCOS are closely related to steroid production (9). Normally, steroid production occurs mainly in the ovaries or adrenal cortex, where cholesterol is converted to progesterone by different cholesterol-producing enzymes, which in turn can be converted to progesterone and androgens, which in turn can be converted to estrogen. These transformation processes require the participation of gonadotropins. For example, LH can regulate the transformation of cholesterol into pregnenolone, and FSH can affect the rate at which aromatase converts androgen into estrogen (10, 11). However, the neuroendocrine dysfunction of the hypothalamic-pituitary-ovarian axis in PCOS patients can affect the normal secretion of LH, FSH, and other hormones and thus, affect the normal production of steroids. Therefore, studies on metabolic dysfunction related-PCOS will help to understand the disease mechanism and develop novel therapies for its treatment.

Bibliometric analysis is based on mathematical and statistical methods to analyze all articles published on a research topic over a period of time, providing an overview of research categories or topics, co-authorship, keyword frequency and the most cited articles or journals, which is exactly what is needed to reveal the frontiers and hotspots of research into metabolic dysfunction in PCOS (12, 13). Previous articles have been published on different aspects related to PCOS, which are coronary heart disease, insulin resistance and infertility (14–16). This is the first bibliometric analysis of PCOS metabolic dysfunction in the last ten years (2012–2021) to investigate the current status of research in this field. First, a linear regression algorithm was innovatively introduced to analyze the number of publications in this field and predict the trend of future publications. We then discussed the collaboration between countries, authors, and institutions in-depth, analyzed the cited journals in a double-graph overlay, and introduced alluvial diagrams to analyze the scientific categories of the articles. In addition, we conducted a comprehensive analysis of keywords and co-cited literature networks.

During the course of this research, we discovered intriguing findings, including a substantial number of citations to articles in the field (80,408) and specifically in 2021 (19,302). Notably, prominent publications such as the Journal of Clinical Endocrinology & Metabolism, OBESITY, and Diabetes Care underscored the significance and relevance of our research topic. Therefore, the objective of this review is to gain a comprehensive understanding of the dynamic changes underlying metabolic dysfunction in PCOS, to assess the notable research advancements made over the past decade as well as the persisting challenges, and to offer valuable insights for future investigations.

## 2 Materials and methods

### 2.1 Data sources and retrieval strategies

In this study, we have used the Web of Science Core Collection (WoSCC), the world’s most comprehensive and influential scientific literature database, to conduct a literature search on metabolic characteristics of polycystic ovary syndrome and collected related articles from the website published till December 25, 2021 (17). The keywords for searching the articles was: TS =((((PCOS)OR(“Polycystic ovary syndrome”))AND (((metabolic*) OR(metabolism*) OR(metabolite*)))). Since this study didn’t include any animal or experiments, ethical consent was not required. The inclusion criteria were limited to “article” and “review”, the language as “English”, and the duration from 2012 to 2021, with the aim of selecting a specific subject and study purpose while also standardizing the language for analysis in the follow-up process. Other literature types, non-English articles, and articles outside this period were excluded. We completed relevant searches and investigated all retrieval records of articles in different formats in plain text format on the same day to generate source files for subsequent use by the different bibliometric analysis software. From the exported articles, we documented the name of the authors, study source, title, keywords, and cited references to avoid errors in retrieving articles at different times.

### 2.2 Methods and statistical analysis

We used the linear regression algorithm of SPSSPRO “Scientific Platform for Professional Statistical Services” to analyze the publication dates of all studies. SPSSPRO is a new online data analysis platform developed by a data analysis team in China, which is different from the traditional Statistical Product Service Solutions (SPSS) and Statistical Analysis System (SAS). Its benefits include simple operation, powerful data processing ability, and accessing various analysis algorithms. There is no record of researchers using SPSSPRO for analytical studies to date. A linear regression algorithm is a statistical analysis method that uses regression analysis in statistics to determine the interdependent quantitative relationship between two or more variables. It is widely used and expressed in the form of y = w’x+e, where e represents the normal distribution of errors with a mean value of 0. The regression analysis conducted in this study focuses on a single independent variable, namely the publication date, and one dependent variable, which is the number of literatures. The relationship between these variables can be approximated by a linear function, known as a univariate linear regression analysis. The specific algorithm employed is based on the method of least squares regression, ultimately resulting in the derivation of a linear regression model (18, 19). The results of the analysis showed that the fitting R^2^ of the model was 0.949, which indicated that the model was excellent. Therefore, the model met the requirements of our study. For collinearity of variables, all large variance inflation factor (VIF) values were less than 10, suggesting that the model has no multicollinearity problem and the model was well constructed. The formula of the model is as follows: Y = –49172.036 + 24.582* year, Y represents the cumulative number of studies within a specific year.

We used VOSviewer1.6.16, Scimago Graphica1.0.15, and CiteSpace5.8.R1 software to analyze the author, source, title, keywords, cited references, and other details of the articles. VOSviewer is a scientific cartographic tool developed by Prof. Van Eck and Prof. Waltman from the Centre for Scientific and Technological Research of Leiden University, for visual bibliometric analysis, which is mainly used to analyze details such as co-authors, countries, and keywords (20, 21). Scimago Graphica, developed in May 2021, is the latest software for browsing, filtering, and visualizing datasets with a simple drag-and-drop feature. Being a code-free tool, it is easy to use and has a fairly versatile application. The CiteSpace was developed by Professor Chaomei Chen of Drexel University in 2004. It can be used to analyze and measure the co-occurrence frequency of key information (keywords, author, region, and citation) in the articles and present the development trend of related studies (22). We used the VOSviewer version 1.6.16 to gather information about the places from where the studies were reported, keywords used in the studies, the total number of publications, quantity, and quality of cited documents, the collaboration between research workers affiliated with different institutions, and the clustering of studies’ keywords. The results obtained from the VOSviewer were further processed using Scimago Graphica 1.0.15 to obtain more sensitive and comprehensive results. The CiteSpace 5.8.R1 was used to understand the pattern of appearance of the keywords and geographical time zones of the published articles. These software applications ensured that the research and development trends of metabolic dysfunction-related PCOS were analyzed from all perspectives.

## 3 Results

Bibliometrics is widely used in the biomedical field and provides a reliable basis for diagnosing and treating various diseases to reasonably predict their future development trend. In this study, through a comprehensive investigation and sorting of the field of PCOS metabolic dysfunction (2012–2021), we found that with an increase in the number of related publications in recent years, the United States has always been the leading contributor to this field, while the cooperation between authors and institutions is complicated. We also identified the four most stable scientific categories in the field over the last decade by introducing alluvial diagrams. Additionally, proteomics, metabolomics, and gut microbiota were the latest research hotspots in this field. Our results are summarized in detail below.

### 3.1 Literature analysis and prediction

**Figure 1:**
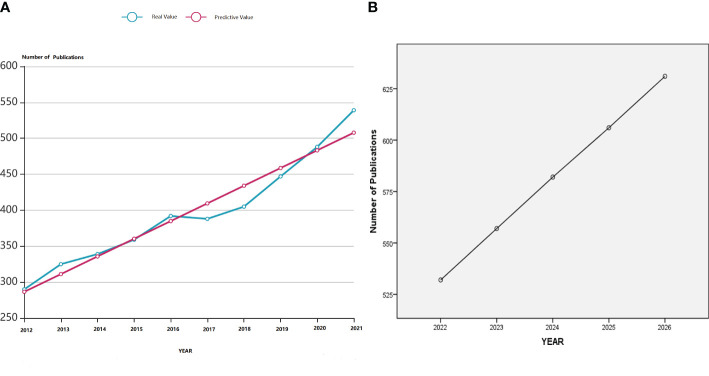
**(A)** A linear regression model showing the number of articles published and the number of articles that were predicted to be published in the last decade on metabolic dysfunction in PCOS. **(B)** Linear regression model for predicting the number of publications in the field of metabolic dysfunction in PCOS in the next five years.

We retrieved 3972 articles from WoSCC and used the linear regression algorithm of SPSSPRO. This statistical analysis suggested that the number of articles published each year, except for the years 2013–2014 and 2016–2017, where there was a slight downward trend, the number of published articles in the last 10 years had increased, especially in the last 5 years. Based on the actual number of articles published each year, the corresponding predicted values were assigned according to the linear regression model formula (**Figure 1A**). In addition, the number of publications for the next five years was predicted from the formula shown in **Figure 1B**. It was seen that the studies on PCOS metabolic dysfunction would continue to increase in the next five years. Although the prediction obtained from the linear regression model might deviate from the actual number of publications in the future, it fairly reflects the research importance on this syndrome. The Web of Science (WOS) citation report showed that the articles were cited 80,408 times; the citation frequency of articles in the past decade was increasing per year. Interestingly, studies published in 2021 were most frequently cited for 19,302 times (**Figure Supplementary Image**).

### 3.2 Contributions and cooperation among the top 15 producer countries

**Figure 2:**
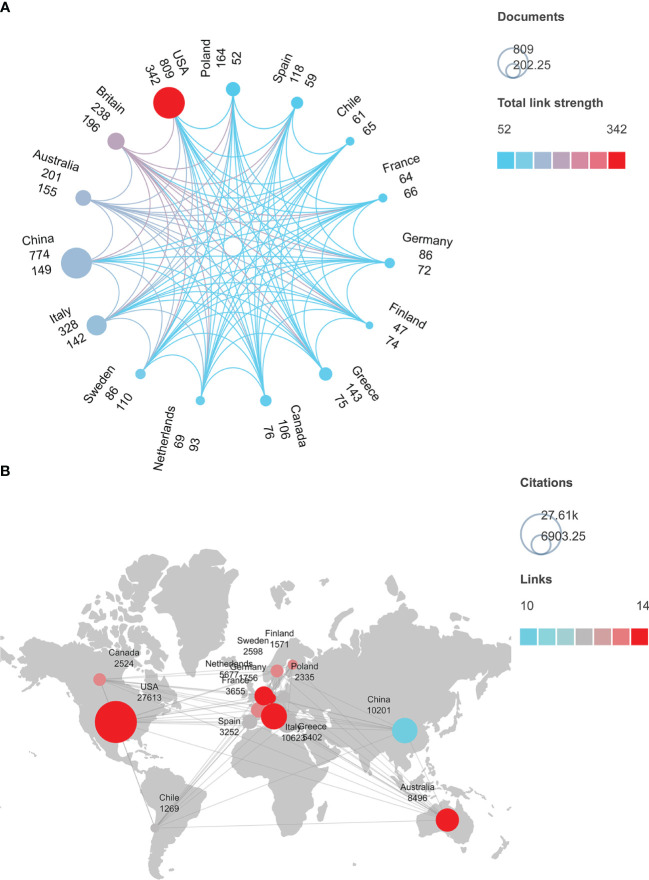
**(A)** Publication volume and cooperation intensity (collaboration network) of 15 countries in the field of metabolic dysfunction in PCOS. **(B)** Citation frequency and the number of collaborations among 15 countries that studied the metabolic dysfunction in PCOS.

We retrieved 3972 articles published in 95 countries around the world. Among these countries, the United States was the topmost contributor with 809 papers; the least number was published by Iraq and Estonia (with only five articles each in the past decade). VOSviewer and Scimago Graphica were used to select the top 15 countries that had collaborations to analyze their publication volume, the total collaborative work between them (**Figure 2A**), their citation frequency, and cooperation between countries (**Figure 2B**). In **Figure 2A**, each circle represents a country, and the size of the circle corresponds to the volume of its publications. This indicates that China (774), Italy (328), Britain (238), and Australia (201) have the most publications in the world after the United States. The color of the circle implies the total intensity of cooperation between the countries, with light blue and red colors representing the lowest and the highest intensity, respectively. Therefore, the total intensity of cooperation between countries increases clockwise from Poland (52) to the United States (342). In **Figure 2B**, the size of the circle signifies the total citations. The top 5 countries with maximum citations are the United States (27,613 times), Italy (10,623 times), China (10,201 times), the United Kingdom (9,281 times), and Australia (8,496 times). The color of each circle indicates the countries having several collaborations; blue indicates the fewest, and red indicates the most. Obviously, in the past decade, China (10) partnered least with other countries to study metabolic characteristics in PCOS, while the United States (14), Italy (14), Australia (14), Germany (14), the United Kingdom (14), and the Netherlands (14) had close cooperation.

### 3.3 Authors and institutions of relevant articles

**Figure 3:**
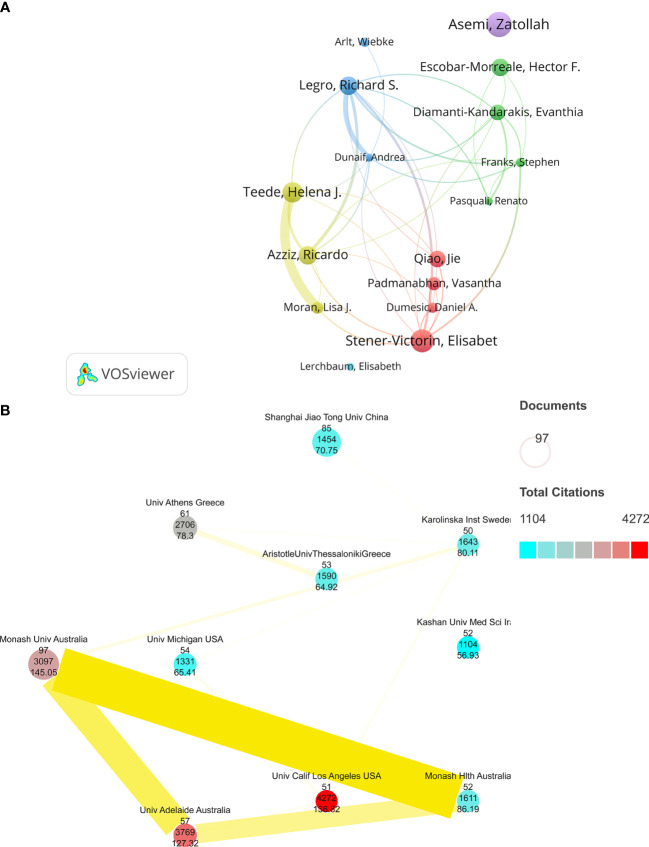
**(A)** A collaborative network of co-authors to study the effect of metabolic dysfunction in PCOS. **(B)** A collaborative network of national institutions on metabolic dysfunction in PCOS.

About 16,088 authors were involved in these studies. Based on co-author analysis by VOSviewer, we defined “core authors” as those who have published at least 15 papers that were cited at least 700 times. Asemi and Zatollah, the two authors, contributed the most in this field with 49 articles published in the last decade; followed by Elisabet Stener-Victorin (44) and Helena J. Teede (39). The articles by Legro and S. Richard were the most frequently cited (3,527 times). The publications by Andrea Dunaif (2364) and Diamanti-Kandarakis and Evanthia (2138) ranked second and third, respectively. In addition, Teede, Helena J., and Moran, Lisa J. have the most cooperative relationship among them. Asemi, Zatollah, and Lerchbaum, Elisabeth are all independent authors without any cooperative relationship with the other 15 authors (**Figure 3A**). A total of 3,635 different institutions conducted studies on PCOS metabolic dysfunction. We used VOSviewer and Scimago Graphica to include only the institutions that had published at least 50 articles that were cited <1100 times; 10 different institutions were thus selected (**Figure 3B**). In **Figure 3B**, the size of the circle represents the number of published articles, and the color represents the frequency of their citations; ten institutions are shown with the average value of citations, and the intensity of the cooperation between institutions is indicated by the width and colors of the lines joining them. Monash University (97) had the most published articles, followed by Shanghai Jiao Tong University (85) and the University of Athens (61). The articles published by the University of California, Los Angeles, were the most cited ones (4,272), while Monash University had the highest average citations (145.0479). Monash Univ and Monash Hlth had the closest cooperation, while Kashan University of Medical Sciences was the only one among the ten institutions that did not collaborate with other institutions (**Figure 3B**).

### 3.4 Cited journals and scientific category analysis

**Figure 4:**
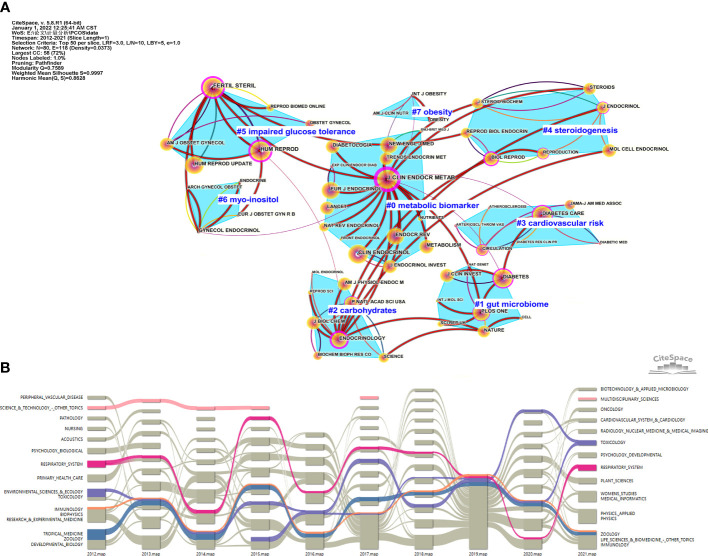
**(A)** Double graph overlay of journals that published articles on metabolic dysfunction in PCOS. **(B)**. The impact map of scientific categories related to the metabolic dysfunction in PCOS.

We assessed the impact of highly cited journals in this research field through CiteSpace. The publications, from January 2020 to December 2021 and January 2012 to December 2021 were analyzed; the first map included the articles published in journals in 1-year out of which we chose the top 50 most cited journals, and the second map included the keywords used for each graph clustering, and finally, the two maps were superpositioned (**Figure 4A**). Each node represents a periodical, the size of the circle represents the number of times the journal was cited, purple halo represents the journal has better centricity (> 0.1). The connection between the two nodes indicates that the two journals under a topic cluster had cooperated for the study; the thick red line attachment suggests the last two years still included the clustering of the theme of the journal. CiteSpace grouped the 50 cited journals into 28 clusters and showed the 8 clusters that collaborated on the study (**Figure 4A**). The #0 indicates that metabolic biomarkers were the largest cluster group, which was also the most concerned topic cluster in many journals in the last two years. The most cited journal was The Journal of Clinical Endocrinology & Metabolism (3471 times). The cluster containing the least number of journals was OBESITY (7#), with only three journals. Obesity and International Journal of Obesity were the only two journals focused on obesity in the last two years. The topic cluster that received the least attention within all journals in the past two years was cardiovascular disease risk (#3), and only Diabetes Care covered this topic (**Figure 4A**). The classification of an article in the Science category was based on the SC field of the Web of Science text set; the Science category can help researchers readily learn the latest work in this research field (23). To show the evolution of scientific categories in the research field related to the metabolic abnormalities in PCOS in the past decade and find out which scientific categories were the most suitable for this field, the alluvial diagram of scientific categories was drawn according to the time trend by CiteSpace. Starting with the relevant science category topics in 2012, the impact map evolves into a pattern of multiple streams flowing smoothly over time through different splintering and merging of science category topics each year until it ends in 2021. In this process, different science categories were merged again. For example, after four years of relatively stable flow evolution, science-technology and other topics were interrupted during the middle years and were finally merged into multidisciplinary sciences. Four of the oldest but continued scientific categories were identified. Over the past decade, the divisions and mergers of each category were color-coded―blue for ZOOLOGY, orange for IMMUNOLOGY, purple for TOXICOLOGY, rose-red for RESPIRATORY SYSTEM. Among these, ZOOLOGY, IMMUNOLOGY, and TOXICOLOGY showed many overlapping splits and mergers over time, proving that the three scientific categories are closely related (**Figure 4B**).

### 3.5 Multi-angle analysis of keywords used in the published articles and co-citation network

**Figure 5:**
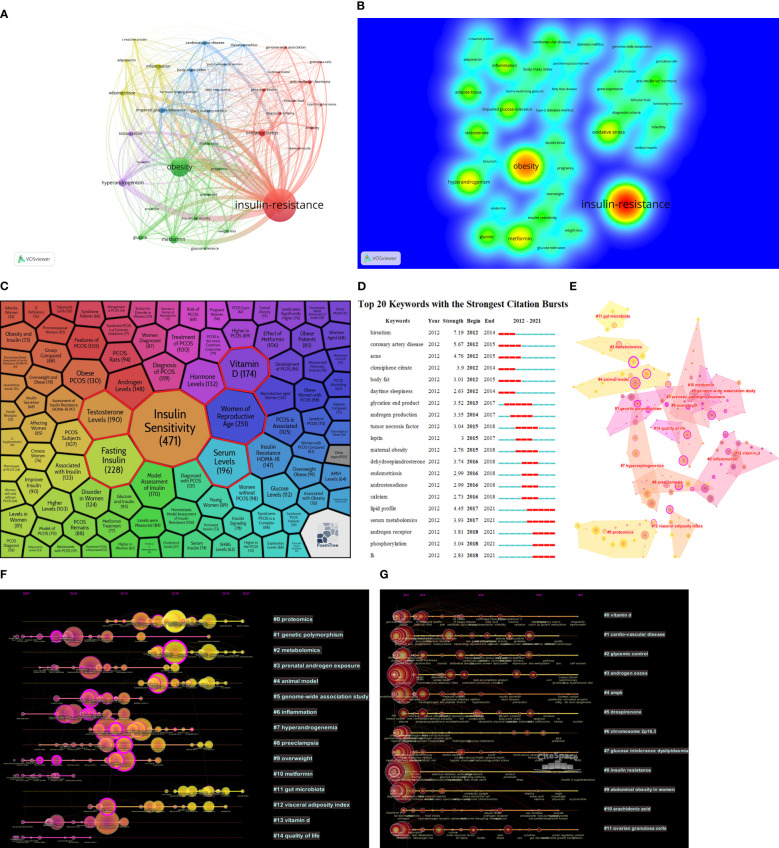
**(A)** Network visualization of keywords used in the published articles related to the topic of metabolic dysfunction in PCOS. **(B)** Heat map of keywords among the published articles on metabolic dysfunction in PCOS. **(C)** Keywords used in the articles in the field of metabolic dysfunction in PCOS. **(D)** Keywords’ appearances in articles related to the study on metabolic dysfunction in PCOS. **(E)** Co-cited network cluster analysis in the domain of metabolic dysfunction in PCOS. **(F)** Co-cited network time zones on metabolic dysfunction in PCOS. **(G)** Time zone map of keywords used in articles on metabolic dysfunction in PCOS.

To assess the main research contents and the change in research perspective over time about the metabolic dysfunctions in PCOS, VOSviewer and CiteSpace were used to draw different visual clustering maps of keywords used in the published articles. After removing redundant keywords such as “PCOS”, “women”, and “patients”, the clustering network visualization and frequency heat map of keywords were created on VOSviewer (**Figures 5A, B**). CiteSpace is connected to the carrot2 system to analyze the key topics and associated top six frequently occurring words; these are highlighted in red boxes and are as follows: Insulin Sensitivity, Women of Reproductive Age, Fasting Insulin, Serum Levels, Testosterone Levels, and Vitamin D (**Figure 5C**). CiteSpace software was used to complete the analysis of the appearance of keywords used in the studies of metabolic dysfunction in PCOS (**Figure 5D**).

CiteSpace software was used to analyze the cluster diagram and time area diagram of co-citation. Total cited articles published over time appear at the top of the image. Different nodes at each level represent the same category; the length of the horizontal line and fore and aft ends are the duration for that category. Each node represents the number of citations, and the size of the peripheral node (purple circles) represents the intermediate centricity (in terms of numerical value). In short, the beginning, popularity, and decline periods of research categories in this field can be obtained by observing the time area chart (24). The co-citation analysis in this research field is divided into 14 different categories (**Figures 5E, F**). Genetic polymorphism (#1) was the most persisting one, which means that the research on metabolic dysfunction during PCOS was mostly based on genetics. Proteomics (#0), metabolomics (#2), and gut microbiota (#11) are the three key topics that were the interest of active research. According to the time zone map of keywords (**Figure 5G**), related studies were conducted continuously in the past decade for other categories except for #1 cardiovascular diseases, #6 chromosome 2p 16.3, and #10 arachidonic acid. This indicates that the pathogenesis of PCOS remains unclear, and further studies from different aspects are needed.

## 4 Discussion

The article analysis software can do scientometric analysis. All the documents on a particular subject under a limited period that contains a variety of effective information are analyzed and quantified in an intuitive way to summarize this effective information. It reveals whether a scientific topic was a hot spot for a certain period in the past, and it is then used to predict the next development trend on that topic. The valid information includes the number of publications and citations and the impact of publications based on the involvement of the countries, institutions, authors, journals, scientific categories, and keywords.

### 4.1 General information study

The comprehensive results indicate that the number of publications and citation frequency on the subject of metabolic dysfunctions in PCOS is on the rise, and it is predicted that it will continue to be an active research topic for the next five years (**Figures 1A, B**; **Figure Supplementary Image**). The United States, China, and Italy are prominent contributors to this research field, accounting for 48.11% of the total number of papers published. The papers published by the United States are the most cited, and they had the most number of research collaborations with other countries, suggesting that the United States had conducted in-depth research in this field (**Figures 2A, B**). Among the top 10 selected institutions, American institutions cooperated mostly with the Australian research institutions; only one institution was from a developing country, while the rest were from developed countries, implying that developing countries started delayed research in this field and need to improve their research outputs (**Figure 3B**).

The assessment and ranking of cited journals can help researchers quickly find the journal suitable for their article submission. It will also become easy to find the topics that most journals in this field are interested in publishing, such as metabolic biomarker was the most discussed topic in this field from 2020 to 2021. Biomarker studies are breakthrough studies that can explore the pathogenesis at the molecular level and provide early warning for diseases, including PCOS (a chronic endocrine disorder) and the investigation of its pathogenesis (**Figure 4A**). The results of the scientific category analysis of the published articles showed a dynamic evolution process of all the scientific categories in the last ten years, and we found the four most stable and suitable scientific categories in the field of PCOS, which were also the focus of the difficult long-term research in this field (**Figure 4B**).

### 4.2 Emerging trends, hotspots, and frontiers

The utilization of literature co-citation networks and keyword clustering can unveil the underlying research structure in the field of metabolic dysfunction associated with PCOS. Through careful examination of these analyses, a wealth of valuable information can be gleaned, including but not limited to luteinizing hormone, serum metabolomics and proteomics, insulin resistance, steroids, multiple complications and therapeutic options. These findings allow for identification of emerging trends and research hotspots within the study of PCOS metabolic dysfunction. In subsequent sections we will discuss their profound significance for this area of research as well as potential implications for future directions.

#### 4.2.1 Gonadotropin research

Abnormal gonadotropin secretion, as one of the common characteristics of PCOS, is a key reference for the clinical diagnosis of the disease. Among them, LH is usually elevated in PCOS, while FSH is mostly at a low level, and its intrinsic pathogenesis is mostly related to neuroendocrine defects (25). The synthesis of LH and FSH is closely related to the GnRH pulse frequency. Experiments on ovaries have shown that the hourly exogenous GnRH pulse frequency can accelerate the secretion of LH, while a 3–4 h pulse frequency is beneficial to the secretion of FSH (26, 27). Other studies on patients with PCOS found that LH increased in response to GnRH, but FSH had no significant response, which was the same as the spontaneous LH pulse frequency increase in PCOS patients (23). Therefore, finding an effective treatment to improve the gonadotropin abnormalities in PCOS patients is necessary. In the latest pharmacological experimental study, saffron petal extract and petal anthocyanin were confirmed to reduce luteinizing hormone and androgen levels in PCOS mice (28). It is confirmed that there is dysregulation of metabolism and oxidative phosphorylation pathways in oocytes of PCOS patients, and metformin can improve such dysregulation and enhance the developmental ability of oocytes in patients with PCOS (29, 30).

#### 4.2.2 Proteomics and metabolomics research

Among 14 different clusters co-cited in studies and were hot topics among researchers accounted #0 from the proteomics field, #2 from metabolomics (**Figures 5E, F**). Various “omics” studies emerging in recent years are tools that analyze large-scale data to better interpret biological research (31). Notably, proteomics and metabolomics have been successively applied to the study of various diseases (32). Proteomics is mainly used to quantitatively analyze protein data obtained from the cells or body fluids using the mass spectrometry method (33). Proteomics applied in the study of metabolic disorders in PCOS was largely to screen out biomarkers (proteins) and understand this disease’s pathological mechanism (34, 35). Metabolomics, a scientific approach, used non-invasive and specific methods to identify potential biomarkers for PCOS in recent years and also elucidated disease pathology (36, 37). Compared with proteomics, which can only show the existence of metabolic disorders in the organism, metabonomics can detect the slightest biochemical changes in the pathophysiology of PCOS, improving the diagnosis methodology for PCOS (38, 39).

#### 4.2.3 Insulin resistance and oxidative stress research

Carrot2 and VOSviewer are also used to identify relevant keywords and find connections between them (**Figures 5A, C**). Prominent words that appear in the articles related to PCOS include insulin resistance, obesity, and androgen overload. The cause of insulin resistance has not been accurately determined, but it may be related to the accumulation of certain lipid metabolites (diacylglycerol and/or neuramide) in skeletal muscle and the oxidative stress-induced pro-inflammatory state (caused by reactive oxygen species). Previous bibliometric analyses have suggested that oxidative stress may exacerbate PCOS and insulin resistance by impairing glucose uptake in musculoskeletal muscle and insulin secretion in pancreatic β-cells, ultimately leading to elevated androgen levels and disruption of the follicular intracellular environment (15). Oxidative stress is caused by excessive production of reactive oxygen species when the balance between pro-oxidant and anti-oxidant in the human body is impaired. Excessive production of reactive oxygen species can cause DNA damage, endothelial damage, and apoptosis of ovarian epithelial cells (40–42). Oxidative stress, induced by ovarian torsion/detorsion (T/D), damages ovarian tissue, including a reduction in follicles, apoptosis of granulosa cells, and an increase in atretic bodies. Excess levels of reactive oxygen species in the ovarian torsion/detorsion process are accompanied by an increase in the Bcl2-Associated X (Bax) protein and a decrease in the B-cell lymphoma-2 (Bcl-2) protein, resulting in ovarian tissue damage (43). Other studies have confirmed that oxidative stress can reduce superoxide dismutase (SOD) and glutathione peroxidase (GPx) activity and result in ovarian T/D, damaging ovarian tissue (44, 45). Additionally, cyclophosphamide can induce apoptosis by increasing reactive oxygen species and causing oxidative stress in ovarian cells, thus, inducing them to initiate programmed cell death and resulting in ovarian tissue damage (46, 47). High levels of various oxidative stress markers were also observed in PCOS, suggesting that oxidative stress might be involved in the pathophysiology of PCOS (48). Some studies have shown that an increase in the level of advanced glycation end (AGE) products in women with PCOS can increase the production of reactive oxygen species, thus, causing oxidative stress in ovarian cells and leading to various negative effects on cell metabolism (49, 50).

#### 4.2.4 Pathological steroid research

As a naturally occurring stereoisomer of inositol, D-chiro-inositol has the potential to safely mitigate multiple metabolic conditions such as PCOS-associated insulin resistance (51–53). The abnormal ratio of D-chiro-inositol to inositol in the ovaries is a common feature of PCOS, and this abnormal ratio might lead to the formation of pathological steroids in PCOS (54). The higher the ratio, the higher the aromatase activity for producing estrogen, while a lower ratio leads to the production of androgens, which are closely related to the generation of PCOS steroids (55). D-chiro-inositol can directly affect the regulation of the steroidase gene in ovarian granulosa cells. This is achieved by reducing the Cytochrome P450 side-chain cleavage (CYP450scc) genes and the aromatase CYP19A1 (56). Studies on steroidogenesis of PCOS have focused on genetics and genes, especially the correlation between ovarian steroids and hyperandrogenemia, mainly including the CYP11A1 gene, the CYP17 gene, the CYP19 gene, and the androgen receptor (AR) gene. These genes mainly encode enzymes or regulate androgen levels in ovarian steroid production (57). Some studies have shown that some loci are related to steroid production in genome-wide association studies (58). DENND1A is not only closely related to PCOS hyperandrogenemia but also influences steroid production by affecting the transcription of CYP11A1 and CYP17 (59). Luteinizing hormone chorio gonadotropin receptor (LHCGR) overstimulates LH by enhancing the expression of the LH receptor, thus, affecting PCOS steroid production (60). Additionally, the elevated level of AGE products in PCOS patients might cause steroid production (49). The specific effects of advanced glycation end products on PCOS steroid production are under investigation. However, its interaction with the membrane receptor late glycation end product receptor (RAGE) can affect the mRNA expression levels of acute regulation of steroidogenesis (StAR), CYP17A1, and 3β-hydroxysteroid dehydrogenase (3β-HSD). The AGE-RAGE signals can affect the formation of PCOS steroids (49).

#### 4.2.5 Research on multiple complications and potential treatment regimens

In a comprehensive evaluation of endometrial function in PCOS, obesity and insulin resistance along with endometrial disorders can result in miscarriage, pregnancy complications, and affect glucose transport; lifestyle changes and taking metformin can improve the function of gestational endometrium in PCOS patients. As for the severely obese person, bariatric surgery is an optimal option (61). Testosterone level indicates hyperandrogenemia; hypertrichosis is the most used clinical diagnostic criterion for hyperandrogenism (62, 63). Excessive androgen affects the metabolic function in PCOS, resulting in long-term metabolic complications, mainly type 2 diabetes, non-alcoholic fatty liver disease, and cardiovascular diseases (64, 65). Adiponectin is a circulating protein produced by adipocytes that are negatively correlated with metabolic disorders. Recently, its collateral homolog C1q tumor necrosis factor-related protein 6 (C1QTNF6) has been shown to intervene in the pathogenesis of PCOS by affecting the inflammatory response in granulosa cells (66, 67).

In addition, several studies have shown that the intestinal microbial community in PCOS patients is different from that of healthy people, especially in their metabolic functions (68–70). The health status of an individual’s metabolism depends on the quantity and diversity of gut microorganisms (71, 72). Thus, metabolic dysfunction in PCOS patients can be treated by improving their gut microbiome. For example, improving intestinal flora, regulating bile acid metabolism, or increasing IL-22 levels can be used for the treatment of PCOS (73). Currently, therapies that can significantly improve the metabolic dysfunction in PCOS patients include fecal microbiota transplantation (61), probiotics (74), and prebiotics (75). It should be noted that while research on the gut microbiota of PCOS patients has been conducted, it remains in its early stages and much of the data and findings are derived from rodent studies (76–78). Therefore, there is currently insufficient evidence to fully elucidate the human gut microbiota and pathogenesis of PCOS, necessitating further human-based studies in this area to better understand the pivotal role played by gut microbiota in PCOS patients. It is necessary to conduct more detailed and systematic studies in this field, focusing on various potential complications of PCOS and the underlying etiology. This can improve the accuracy of early diagnosis and the effectiveness of the treatment of PCOS.

### 4.3 Practical implication

The practical significance of this study encompasses three main aspects: (1) Junior researchers can efficiently acquire essential knowledge on PCOS metabolic dysfunction by studying the research findings of prominent authors and institutions presented in this paper, thereby enhancing their scientific research proficiency and capabilities. (2) By utilizing journal overlay analysis, we can identify relevant research hotspots and suitable journal titles, which will greatly facilitate future scholars working in the same field. (3) Through co-citation analysis and keyword co-occurrence analysis, we can effectively identify the current research trends and unresolved issues related to PCOS metabolic dysfunction. This enables us to formulate targeted policies as future research goals and allocate financial resources towards practically addressing the challenges faced within this research domain.

### 4.4 Limitation

Although this study provides the first comprehensive bibliometric analysis of metabolic dysfunction in PCOS, there are several limitations that may impact its findings. Firstly, the data used in this article were obtained solely from the WOSCC database, which may have excluded some valuable information. Secondly, only articles and reviews were included while political and social publications such as editorials and books were not considered. Thirdly, although our search strategy was designed to be comprehensive, it is possible that other relevant keywords were overlooked which could affect our results. Lastly, due to the vast vocabulary involved when manually combining synonyms errors are inevitable. However, given the large amount of data generated by numerous publications included in this study any potential bias should be minimal.

## 5 Conclusion

Several bibliometric analysis software was used to analyze the status of the field of research on metabolic dysfunction in PCOS from different perspectives. This study not only contains information on countries, journals, authors, institutions, and scientific categories but also specifically illustrates the internal relationship between keywords in different clustering groups and their influence on this field. Furthermore, we found that the main research topics to be investigated in the future for a better understanding are lipid profile, androgen receptors, phosphorylation, luteinizing hormones, proteomics, metabolomics, and gut microbiota. This bibliometric analysis might provide more answers to researchers involved in this field.

## Author contributions

Conceptualization: TC and YX. Validation: JR, YX, and TC. Data curation: YX and ZC. Writing—original draft preparation: YX. Writing—review and editing: TC. Visualization: JR. Supervision: JR and YX. Project administration: JR and TC. Funding acquisition: JR and TC. All authors have read and agreed to the published version of the manuscript.

## Supplementary material

Supplementary Figure Trends in citation frequency of articles related to metabolic dysfunction in PCOS over the last decade.
