# Supplementary figures and images for: Trends in metabolic dysfunction in polycystic ovary syndrome: a bibliometric analysis
Source: Front Endocrinol (Lausanne). 2023 Aug 28;14:1245719. doi: 10.3389/fendo.2023.1245719 (PMC10494444; doi:10.3389/fendo.2023.1245719)

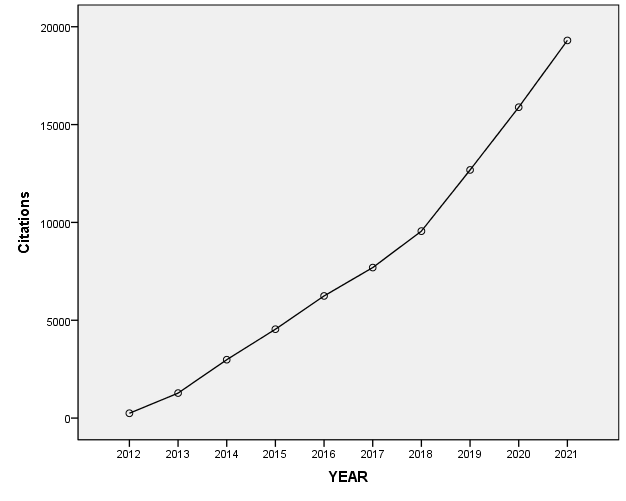

Supplement: Supplementary Figure — Trends in citation frequency of articles related to metabolic dysfunction in PCOS over the last decade. [file Image_1.tiff]
